# Near IR fluorescent conjugated poly(ethylene glycol)bisphosphonate nanoparticles for in vivo bone targeting in a young mouse model

**DOI:** 10.1186/s12951-015-0126-0

**Published:** 2015-11-14

**Authors:** S. Rudnick-Glick, E. Corem-Salkmon, I. Grinberg, R. Yehuda, S. Margel

**Affiliations:** Department of Chemistry, The Institute of Nanotechnology and Advanced Materials, Bar-Ilan University, 52900 Ramat Gan, Israel; The Mina and Everard Goodman Faculty of Life Sciences, Bar-Ilan University, 52900 Ramat Gan, Israel

**Keywords:** Bisphosphonate, Nanoparticles, Targeted delivery, Near-IR fluorescent

## Abstract

Bisphosphonate (BP) compounds are widely used in the treatment of bone disorders. This group of drugs with a high affinity to Ca^+2^ ions is rapidly attracted to bone mineral, especially in areas of high resorption. We have engineered unique biodegradable BP nanoparticles (NPs) by dispersion co-polymerization of the monomers methacrylate-PEG-BP) and (3-Aminopropyl)mathacrylamide) with the crosslinker monomer tetra ethylene glycol diacrylate. These NPs possess a dual functionality: (1) covalent attachment of a dye (e.g. near IR dye) or a drug to the nanoparticles through the primary amine groups on the surface of the NPs; (2) chelation to the bone mineral hydroxyapatite through the BP on the surface of the NPs. This study describes the uptake of the unique near IR fluorescent Cy 7-conjugated BP NPs in bone of a young mouse model. Blood half-life studies revealed a relatively long half-life (approximately 5 h) due to a high concentration of PEG in the BP NPs as well as a relatively long whole body clearance (approximately 2 weeks). Body distribution studies showed a specific uptake of the BP NPs in bone. These unique engineered BP NPs are planned to be utilized in future work for diagnostic and drug delivery systems that are targeted to bone disorders.

## Background

**Fig. 1:**
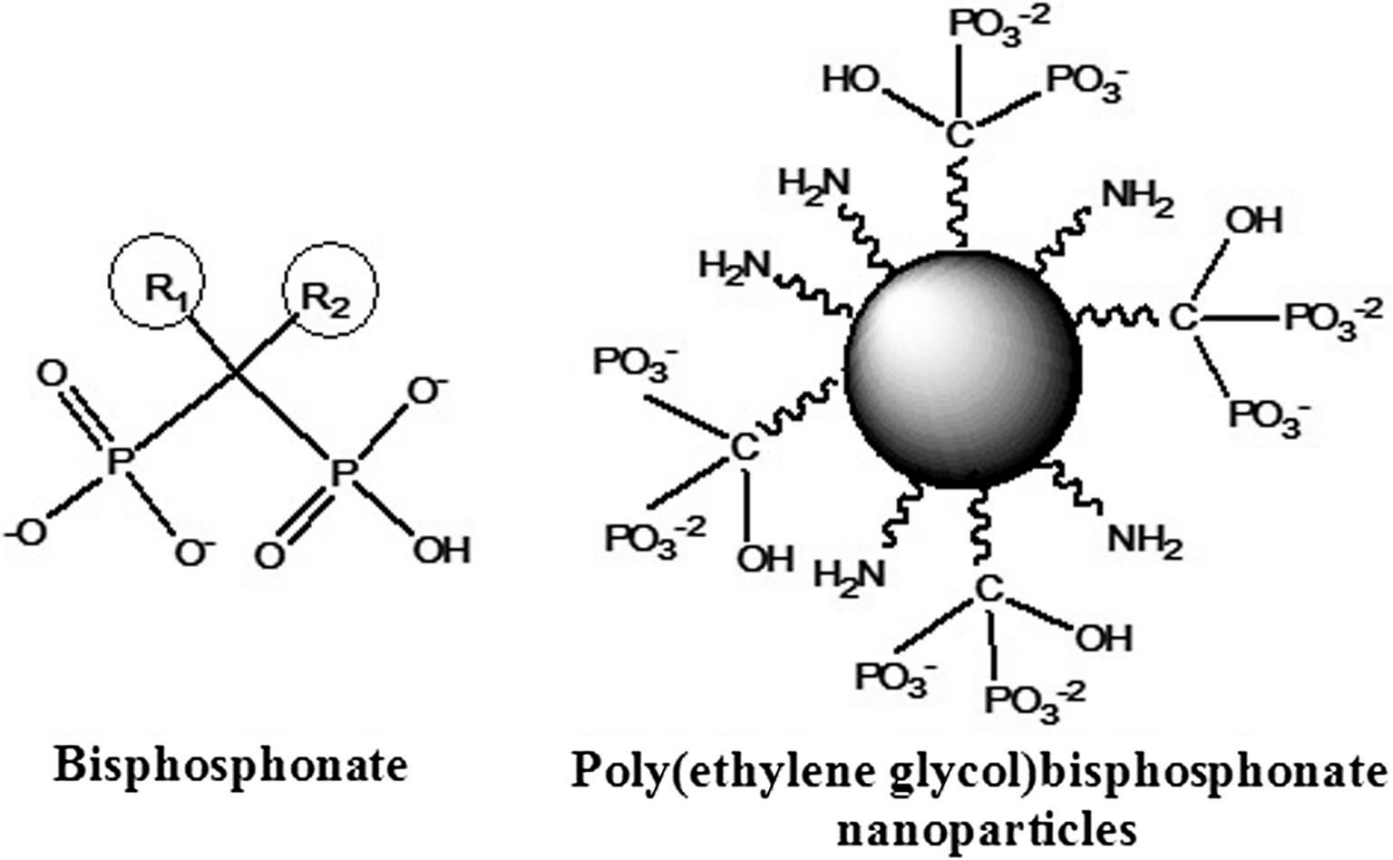
BP compared to the novel poly(ethylene) BP NP which consists of multiple BP functional groups and primary amines per particle. The free amine groups on the surface are available for the conjugation of a dye/drug

Bisphosphonates (BPs) are widely used in the treatment of bone diseases such as osteoporosis [1, 2], Paget’s disease [3, 4] and various cancers [5, 6]. This group of drugs, due to their high affinity to Ca^+2^ ions, rapidly localizes to bone mineral, especially in areas of high resorption [3]. BP bind to hydroxyapatite (HAP) either through a bidentate chelation or through a tridentate chelation, which is attributed to the two phosphonate groups and the hydroxyl side chain (R_1_) [3]. BP, as opposed to pyrophosphate (PPi), the endogenous analog, is resistant to hydrolysis due to the presence of the P–C–P bond (Fig. 1).

At the resorption site, BPs inhibit osteoclast activity through an intracellular pathway, which is associated with the R_2_ side chain. The bioactivity of BPs can be described either as simple BP (non-nitrogen containing BP) or as nitrogen containing BP (N-BP). The simple BPs mimic PPi by metabolically integrating into non-hydrolysable analogues of ATP. On the other hand N-BPs inhibit the mevalonate biosynthetic pathway [3, 7].

BPs are administered orally or intravenously [8]. Oral absorption of BPs is relatively poor (<1 %) whereas following IV administration approximately 50 % is integrated into the bone. BPs are eliminated via the renal system. The BPs taken up by the bone remain in situ for a long period, depending on the rate of the bone remodeling cycle, and are released back into the blood stream [8].

Polyethylene glycol (PEG) is a non-toxic, biocompatible, non-immunogenic polymer, and is widely used in biopharmaceuticals to improve pharmacokinetics. PEGylation modifies the physical and chemical properties of the drug, and can decrease immunogenicity and improve drug solubility. In addition PEGylation improves drug stability. Consequently, it extends blood half-life and therefore reduces dose frequency [9, 10].

In the past few years, near-infrared (NIR) fluorescent imaging has attracted much interest for its potential use in in vivo imaging applications. NIR fluorescence (700–900 nm) exhibits low auto-fluorescence and higher penetration, compared to UV and visible light, due to lower light scattering by the biological tissue at this wavelength [11, 12].

A variety of nanoparticles (NPs) containing NIR fluorescent dyes have been developed, including those based on silica, calcium phosphate and lipoprotein [13, 14]. They exhibit many advantages over free NIR dye. These include enhanced photostability and biocompatibility. Due to the large surface area of the NPs many functional groups are available to the conjugation of a relatively large number of dye molecules thus leading to a significant improvement of the fluorescent signal.

In this work we describe the use of a novel bone imaging NP which utilizes BP functional groups (Fig. 1) in order to target areas of high resorption. In a previous study by our group we demonstrated that these biodegradable poly(ethylene glycol) BP NPs (BP NPs) possess a similar inhibition activity to Alendronate [10] and hence could be considered a suitable bone targeting agent. The functional cross-linked BP NPs of a narrow size distribution, with a dry diameter of 43 ± 5 nm and a hydrodynamic diameter of 160 ± 13 nm [15], were synthesized by heterogeneous dispersion co-polymerization of the new BP monomer MA-PEG-BP (methacrylate-PEG-BP) [10] with the monomer APMA (3-Aminopropyl)mathacrylamide) and the crosslinker monomer TTEGDA (tetra ethylene glycol diacrylate). The monomer APMA contains a primary amine which allows for the covalent binding of a dye to the surface of the particles. For optical imaging of areas of high bone resorption, the NIR dye (Cy 7) was covalently attached to the surface of these BP NPs [15]. In ovo imaging on a chicken embryo model clearly indicated the affinity of these BP NPs to the chicken embryo bones [15].

Due to BP NPs high affinity to areas of active bone turnover these NPs can be utilized in the diagnostics and therapy of a variety of bone disorders, which are characterized by high exposure of calcium ions, e.g. osteoporosis, primary and metastatic bone cancer, Paget’s disease. In this study we investigated the in vivo imaging application of these NIR fluorescent Cy 7-conjugated BP NPs. The study was carried out on a 4 week old mouse model which was at the stage of rapid bone development. We followed the body distribution and whole body clearance, and evaluated the half-life of the NIR fluorescent Cy 7-conjugated BP NPs. In particular the BP NPs bone targeting ability and retention was studied.

## Methods

### Chemicals

The following analytical-grade chemicals were purchased from commercial sources and used without further purification: polyethylene glycol methacrylate (MA-PEG, Mn 360), tetraethylene glycol diacrylate (TTEGDA), polyethylene glycol methacrylate ether (MA-PEG-OCH_3_, Mn 300), potassium persulfate (PPS), O-[(N-Succinimidyl) succinyl-aminoethyl-O′-methylpolyethylene glycol (PEG-NHS, Mw 750), polyvinylpyrrolidone (PVP, Mw 360 K), sodium hydroxide (NaOH, 1 N), hydrochloric acid (HCl, 1 N), anhydrous dichloromethane, anhydrous N,N-dimethylformamide (DMF), chromium oxide, isopropanol, magnesium sulfate (97 %), triethylamine (99 %), methanesulfonyl chloride, sodium chloride, sodium azide (99.5 %), triphenylphosphine, glycine and O,O′-Bis[2-(N-succinimidyl-succinylamino)ethyl]polyethylene glycol (NHS-PEG-NHS, MW 3,000) from Sigma (Rehovot, Israel); N-(3-aminopropyl) methacrylamide hydrochloride, (APMA) from Polysciences, Inc. (Warrington, PA, USA) Dialysis membrane (1000 K–16 MM), bicarbonate buffer (BB, 0.1 M, pH 8.4), sodium carbonate and sodium bicarbonate (BB) from Bio-Lab Ltd. (Jerusalem, Israel); Cy 7-NHS ester from Lumiprobe Corporation (Florida, USA); Dulbecco’s phosphate-buffered saline (PBS) from Biological Industries (Bet Haemek, Israel). Water was purified by passing deionized water through an Elgastat Spectrum reverse osmosis system (Elga Ltd., High Wycombe, UK).

### Synthesis of MA-PEG-BP

The monomer MA-PEG-BP was prepared as described in the literature [10]. Jones Reagent was prepared by adding H_2_SO_4_ (900 μL) to an ice-cooled vial containing CrO_3_ (900 mg), followed by addition of distilled water (2.7 mL). The orange mixture was stirred to homogeneity. MA-PEG-OH (1, 1.95 mL, 6 mmol) was stirred with dry acetone (30 mL) and cooled in an ice-bath. Jones Reagent (3.6 mL, 1.5 eq.) was then added dropwise. The mixture was stirred while cooling for 30 min and then left to warm to room temperature (rt) and stirred at rt overnight. i-PrOH (2 mL) was added. The mixture was stirred for 1 h and then evaporated to remove acetone and i-PrOH. The resulting aqueous solution was extracted three times with chloroform. The combined organic phases were dried over Na_2_SO_4_, followed by evaporation to obtain MA-PEG carboxylic acid as a slightly yellow liquid.

MA-PEG carboxylic acid (DM-10, 750 mg, 2 mmol) was dissolved in dry dichloromethane (5 mL). DMF (1 drop) was added as catalyst, followed by oxalyl chloride (340 μL, 4 mmol, 2 eq.). Gas evolution was observed, the mixture was stirred at rt overnight and gradually turned orange. The resulting mixture was evaporated to dryness, yielding an orange oil which was dissolved in THF (5 mL). Tris(trimethylsilyl)phosphite (1.34 mL, 4 mmol, 2 eq.) was added and the mixture was stirred at rt for 1 h and then evaporated to dryness. Methanol (MeOH) (5 mL) was added stirred overnight at rt. NaOH in MeOH (240 mg, 6 mmol) was added to the mixture. A white precipitate was obtained and dried in a desiccator overnight.

### Synthesis of the BP NPs

BP NPs were prepared as described in the literature [16]. MA-PEG-BP 45 mg, APMA 5 mg and TTEGDA 50 mg (total monomers concentration was 5 % w/v) were added to a vial containing 8 mg of PPS (8 % w/w) as initiator and 20 mg PVP 360 K (1 % w/v) as stabilizer, dissolved in 2 mL of 0.1 M BB. The vial containing the mixture was purged with N_2_ to exclude air and then shaken at 83 °C for 8 h. The BP nanoparticles obtained were washed to remove excess reagents by extensive dialysis cycles (cut-off of 1000 k) with purified water.

### Synthesis of the NIR fluorescent Cy7-conjugated BP NPs

NIR fluorescent BP NPs were synthesized as describe in the literature [16]. NIR fluorescent BP NPs were prepared by a reaction of the primary amino groups on the BP NPs with Cy7-NHS ester. Cy7-NHS ester (2 mg) was dissolved in 0.5 mL of anhydrous DMSO. 250 µL of the Cy7-NHS ester solution was then added to 5 mL of the BP NPs dispersed in 0.1 M BB (2 mg/mL), and the reaction was stirred for 1 h at rt. Blocking of residual amine groups was then accomplished by adding 0.5 mg of PEG-NHS. The reaction was then stirred for 30 min at rt. The NIR fluorescent-conjugated BP nanoparticles obtained were washed to remove excess reagents by extensive dialysis in purified water (cut-off of 1000 k).

NIR fluorescent control nanoparticles possessing OCH_3_ groups instead of the BP groups were prepared similarly, substituting the monomer MA-PEG-BP for MA-PEG-OCH3.

### Animal experiments

All experiments in the research were conducted under a protocol approved by the Institutional Animal Care and Use Committee at Bar-Ilan University. All mice were weighed prior and throughout the experiments (20–25 g).

#### NIR fluorescent Cy7-conjugated BP NPs bone targeting and body distribution in female BALB/C mice

Bone targeting and body distribution studies were carried out on a 4 week old female BALB/C mouse model (Harlan Laboratories, Inc. Israel). Mice were anesthetized by intraperitoneal injection of Ketamine (40–80 mg/kg body weight) and Xylazine (5–10 mg/kg body weight), and their fur shaved with an electric hair clipper as necessary throughout the experiment. 100 µL Cy 7-conjugated BP NPs and control NPs (0.1 mg/ml) suspended in PBS were IV injected via the tail vein. Mice treated with PBS were used as a negative control group for assessment of tissue autofluorescence and background signals. Whole body, bone, blood, kidney, liver, spleen, brain and heart images were studied by the Maestro II in vivo imaging system, for 2D planar fluorescence imaging of small animals (Cambridge Research and Instrumentation, Inc., Woburn, MA, USA). A NIR excitation/emission filter set was used for our experiments (λ_ex_: 710–760 nm, λ_em_ > 750 nm). The liquid crystal tunable filter (LCTF) was programmed to acquire image cubes from λ = 790–860 nm with an increment of 10 nm per image. Tissue autofluorescence and undesired background signals were removed by spectral analysis and linear unmixing algorithm. Fluorescence intensity measurements were performed using ImageJ software.

## Results and discussion

### BP NPs synthesis and characterization

The non-toxic biodegradable functional cross-linked polymeric BP NPs were synthesized by heterogeneous dispersion co-polymerization of the new BP monomer MA-PEG-BP (which provides the targeting functional group) with the monomer APMA (containing a primary amine which allows for the covalent binding of a dye to the surface) and the crosslinker monomer TTEGDA. These NPs are biodegradable due to the presence of ester and amide bonds which can be enzymatically cleaved. As both MA-PEG-BP and TTEGDA contain a PEG component it can be postulated that the NPs possess a relatively long half-life [9]. These BP NPs have a dry diameter of 43 ± 5 nm, as analyzed using electron microscopy (TEM) and a hydrodynamic diameter of 160 ± 13 nm as measured by DLS, which concurred with previously published measurements [15]. The mole ratio between BP (belonging to the MA-PEG-BP monomer) and the amine groups (belonging to the APMA monomer) in the particle was analyzed by elemental analysis (Perkin-Elmer 2400 series II Analyzer). The initial mole ratio of [MA-PEG-BP]:[APMA] prior to polymerization was 4:1, however after polymerization the ratio was found to be 2.8:1. The reduction in the amount of the MA-PEG-BP monomer polymerized could be due to the steric effect of the charge on the BP monomer [16]. The NIR fluorescent dye Cy 7 was covalently attached to the surface of these BP NPs via the primary amine groups for in vivo imaging of the body distribution, clearance and bone affinity of the BP NPs. Since the initial concentration of Cy7-NHS was much higher than that of the –NH_2_ surface concentration, we assume that all the surface available primary amine groups interacted with Cy7-NHS, so that the surface mole ratio of BP to Cy7 is 2.8:1. DLS measurements of Cy7-conjugated BP NPs exhibited a similar hydrodynamic diameter to the non-conjugated BP NPs.

### Whole body clearance of IV injected Cy7-conjugated BP NPs in a young mouse model

Increased bone resorption is characteristic of a variety of bone disorders, including Paget’s disease, osteoporosis, and primary and secondary bone cancer. Bone remodeling involves both resorption and formation [17]. In this study, 4 week old female BALB/C mice were used to demonstrate active bone resorption, as mice at this age exhibit rapid skeletal development [18].

**Fig. 2:**
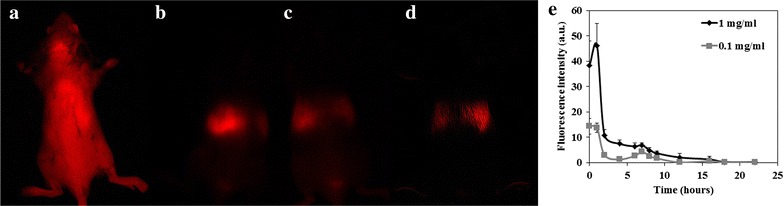
Whole body clearance of the NIR fluorescent Cy7-conjugated BP NPs. 100 µL of 0.1 mg/mL of cy7-conjugated BP NP were injected via the tail vein, and the mice were exposed for 1000 ms (λ_ex_: 710–760 nm, λ_em_ > 750 nm) using the Maestro in vivo imaging system. Spectrally unmixed, high contrast whole body images were obtained after t = 0 (**a**), t = 1 week (**b**), t = 2 weeks (**c**) and t = 4 weeks (**d**). Schematic whole body fluorescent clearance (**e**)

The whole body clearance was investigated at two different concentrations of the Cy7-conjugated BP NPs. Cy7-conjugated BP NPs suspended in PBS (0.1 and 1 mg/mL) were IV injected (100 µL) via the tail vein and were scanned once a week for 22 weeks (n = 10 mice per sub group). Fur was removed at each imaging point to prevent the signal being obscured. The whole body fluorescence was exposed for 1000 ms using the Maestro II in vivo imaging system and fluorescence intensity measurements of the whole body were performed using ImageJ software. The spectrally unmixed, high contrast whole body image, 1 week post injection (Fig. 2), was found to be similar to initial scanned body image immediately following injection, illustrating the retention of a high concentration of the Cy7-conjugated BP NPs. At 2 weeks post injection, there was a marked decrease in the whole body fluorescence (79 and 72 % decrease respectively), demonstrating a significant clearance of the NPs. Fluorescence continued to slowly decrease and at 4 weeks post injection a 91 and 80 % decrease respectively was measured and appeared to be predominantly concentrated in the hepatic region, as well as in the legs. This possibly indicates the incorporation of the BP NPs into the structure of the developing bone which would eventually be released during the natural course of bone remodeling [3, 8]. After 22 weeks 99.5 % decrease was observed at both concentrations of Cy7-conjugated BP NPs.

### Blood half-life of Cy7-conjugated BP NPs

**Fig. 3:**
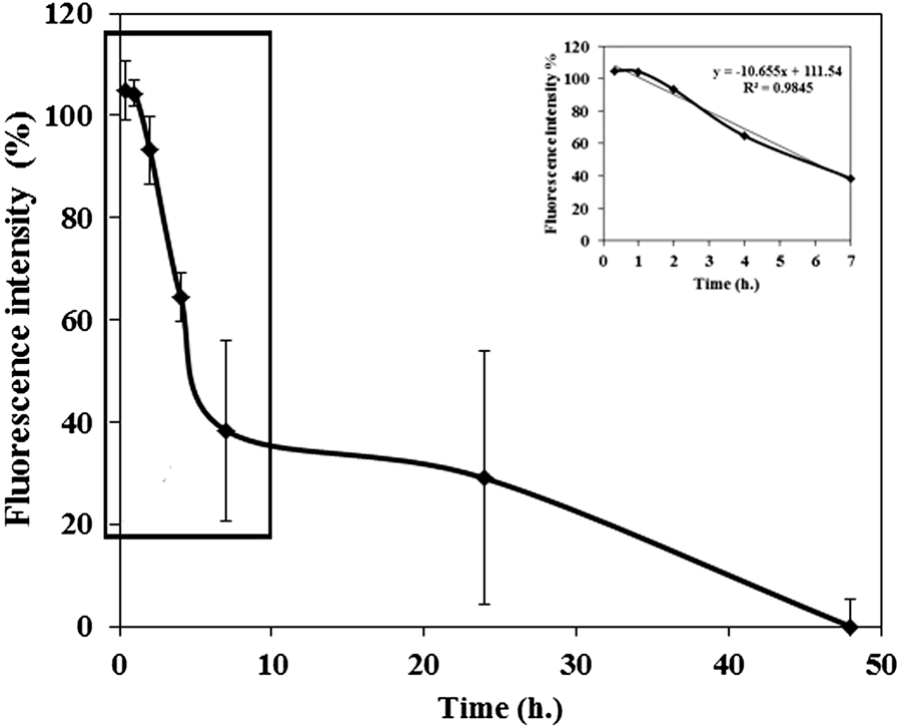
Blood half-life of Cy 7-conjugated BP NPs. 100 µL of Cy7-conjugated BP NP (1 mg/mL) were IV injected via the tail vein and blood was drawn at different time intervals (n = 6). Insert shows the linear fit y = −10.655 + 111.54 with a R^2^ value 0.9845 for the blood half-life

Most BPs have a half-life of around 1–2 h and are eliminated rapidly from the plasma in both animals and humans [1]. NPs also exhibit a relatively short half-life which is dependent on particle size [19]. Fang et al. demonstrated that the blood clearance of the small PEGylated NPs (<100 nm) was slower than that of larger NPs (~200 nm) with a similar formulation [20]. In order to determine the PEG containing BP NPs half-life, 100 µL Cy7-conjugated BP NPs at concentrations of 0.1 and 1 mg/ml were IV injected via the tail vein of 4 week old female BALB/C mice (n = 6). Blood was drawn at 0, 20 min, 1, 4, 7, 24 and 48 h post injection. The blood half-life of the Cy7-conjugated BP NPs was calculated according to a linear trend line based on the first 7 h post injection (Fig. 3). The coefficient used was y = −10.655 + 111.54 with a R^2^ value 0.9845, indicating a very good correlation. Blood half-life was found to be approximately 5 h. This is a relatively long half-life both for BPs [1] and NPs [21] and can be attributed to the high concentration of PEG [21–23] in the BP NPs.

### Soft organ body distribution of Cy7-conjugated BP NPs

**Fig. 4:**
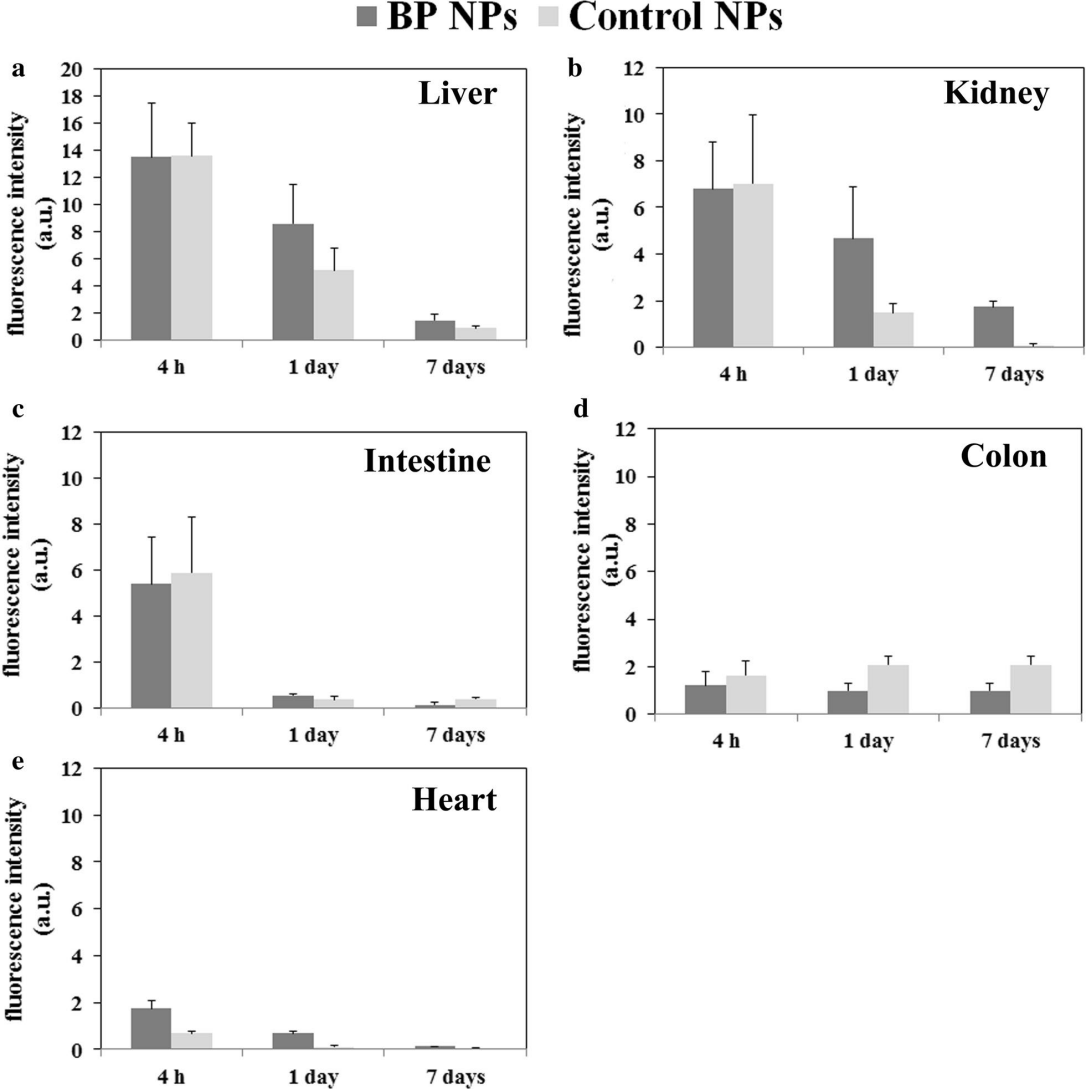
Body distribution of Cy7-conjugated BP NPs compared to control NPs. Mice were injected with 100 µL of 0.1 mg/mL of NPs, were sacrificed 4 h, 1 and 7 days post injection and organs were harvested. Liver (**a**), Kidney (**b**), Intestine (**c**), Colon (**d**) and Heart (**e**). Spectrally unmixed, high contrast organ images were obtained and fluorescence intensity measurements of the individual organs was performed using ImageJ software

The reduction in the NIR fluorescence signal of the whole body image was then further studied. A comparison was made between mice injected with Cy7-conjugated BP NPs at a concentration of 0.1 mg/mL and mice injected with a similar concentration of control NPs, which did not contain the BP functional groups. Mice (n = 9) were sacrificed after 4 h, 24 h and 7 days post IV injection. Several organs were extracted and their fluorescence measured using the Maestro II in vivo imaging system and fluorescence intensity measurements of the individual organs were performed using ImageJ software. The uptake of the fluorescence differed greatly between liver and other extracted organs and therefore exposure times were adjusted accordingly. For liver, exposure time was 5000 ms and for the rest of the organs, 10,000 ms. Hence the results obtained compare the rate of clearance of both the BP and control NPs by the individual organs and not a comparison of the uptake by the liver of the BP and control NPs, to the other organs. Whilst the liver, kidney, heart, lungs and spleen clearance of the Cy7-conjugated BP NPs in general showed a similar trend to the control NPs, there were some discernable differences. All organs showed an initial uptake after 4 h followed by a decrease in fluorescence after 1 day and a further decrease after 7 days post injection (Fig. 4). No fluorescence was seen in the brains extracted hence may indicate that the BP NPs do not cross the blood brain barrier.

The liver exhibited a higher fluorescent intensity at lower exposure time after injection for both the BP NPs and the control NPs compared to the other extracted organs. This could be explained by the PEGylation, which increases NPs stability, and consequently necessitates hepatic degradation and excretion via the liver, into bile and feces. Uptake of NPs by the liver may be rapid but the degradation and biliary excretion is slow [24, 25]. The slow biliary excretion could also explain the fluorescence in the colon which did not decrease. On the other hand, the kidney fluorescence could be explained by the enzymatic cleavage of amide and ester bonds, which release the Cy7, or by break down of the NPs. The free Cy7 (or degraded fragments of the NPs with Cy7 still attached) are small molecules and can be excreted by the kidney.

### Bone targeting of Cy7-conjugated BP NPs

**Fig. 5:**
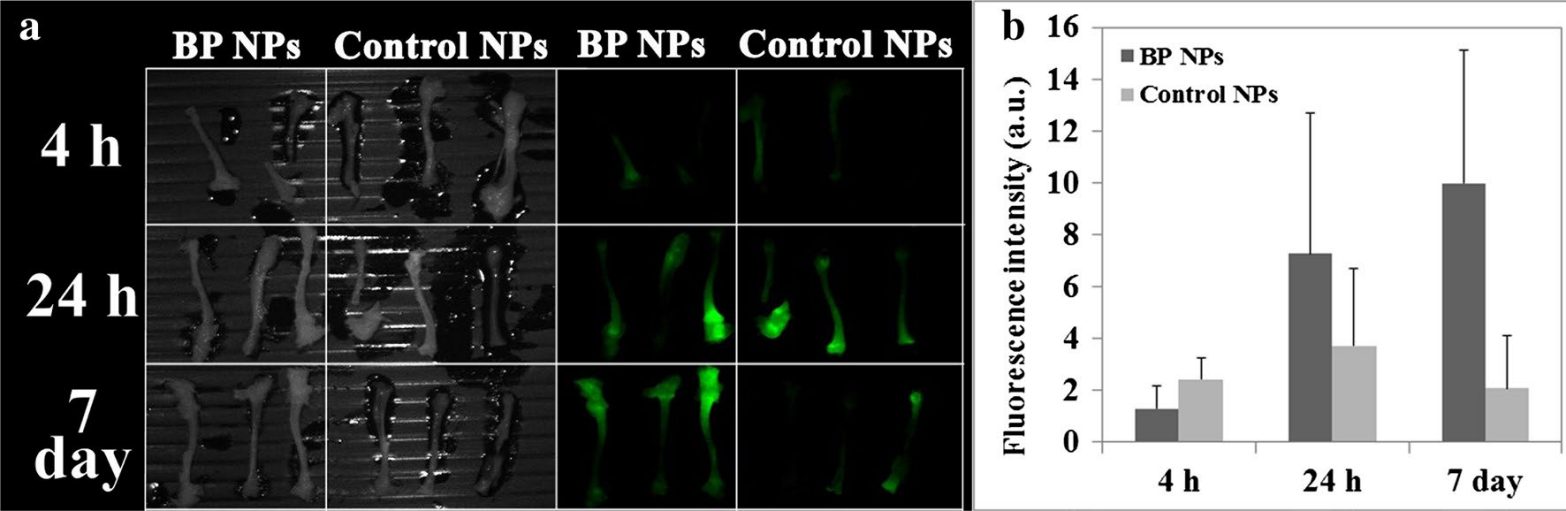
Bone targeting of Cy7-conjugated BP NPs. Mice were treated with 100 µL of 0.1 mg/mL of Cy7-conjugated BP NPs. **a** NIR fluorescent images of the bones extracted from mice treated with cy7-conjugated BP NPs and control NPs. **b** Histogram of the fluorescence of the bones treated with cy7-conjugated BP NPs and control NPs. Spectrally unmixed, high contrast bone images were obtained and fluorescent intensity measurements was performed using ImageJ software

When comparing the fluorescence of Cy7-conjugated BP and control NPs in bone, there is a marked difference in the findings (Fig. 5). 4 h post injection, both NPs exhibit a similar fluorescent intensity. However, 24 h post injection, the fluorescence intensity of bone treated with BP NPs exhibited a marked increase, whereas the fluorescence of bone treated with the control NPs remained unchanged. 7 days post injection, the BP NPs remain at a high concentration in the bones and may indicate their incorporation into the newly formed bone.

Bone growth is associated with an enhanced blood supply to the area and the accompanying bone remodeling involves exposure of HAP, factors which lead to an accumulation of BP [1, 26]. Our NIR fluorescent images demonstrate that the fluorescence is concentrated at the end of the bones, the main areas of active bone growth. Hence we may conclude that due to the high concentration of BP in the NPs, bone in a state of active turnover is preferentially targeted by the Cy 7-conjugated BP NPs.

## Conclusions

In this research we investigated the uptake of Cy 7-conjugated BP NPs in bone in a young mouse model.

The whole body clearance of the Cy 7-conjugated BP NPs was followed and it was found that after 2 weeks the BP NPs where present at a very low concentration. The blood half-life of the Cy 7-conjugated BP NPs was calculated at approximately 5 h, which is a relatively long half-life for both BP and control NPs [1, 21]. This is attributed to the incorporation of PEG in the BP NPs. These results are consistent with previous studies that indicated PEGylated NPs (<100 nm) have a relatively long blood half-life and an enhanced permeability and retention (EPR) effect, hence showing the importance NPs size and surface composition in achieving effective, targeted delivery [19].

On reviewing the results of soft organ uptake, in comparing the clearance of Cy 7-conjugated BP NPs with the control NPs, no significant differences were detected between the harvested organs. The highest concentration of both NPs was found in the liver. In addition, high concentrations were also found in the kidneys and colon. The high colonic concentration may be associated with biliary excretion of the NPs into the GI tract [24, 25], and their elimination via feces, hence, the continuous fluorescence of the colon. On the other hand, the kidney fluorescence could be explained by the metabolism of the Cy 7-conjugated NPs, resulting in the cleavage of the amide bond. The free Cy7 is a small molecule and can therefore be excreted by the kidney.

However, bone differed from the other harvested organs. In bone the Cy 7-conjugated BP NPs did not decrease over time. The accumulated Cy 7-conjugated BP NPs exhibited a high and stable fluorescent concentration due to the high affinity of BP to exposed HAP in developing bone.

This mouse model, whilst confirming previous findings has limited application for the long term study of the targeting of BP NPs to bone resorption sites. The age of the mice at the start of experiment and the length of the rapid growth period restricts the time available for study [27]. Poly(MA-PEG-BP) NPs prolonged half-life and preferential uptake in areas of high bone activity provide exciting potential for these NPs to be utilized in diagnostic and drug delivery systems that are targeted to bone. Future plans include further investigation of the bone targeting ability of these BP NPs on more suitable mouse models, such as osteoporosis and osteosarcoma models, and to explore their potential as a drug delivery system in different bone disorders.

## Abbreviations

BP: bisphosphonate
HAP: hydroxyapatite
PPi: pyrophosphate
ATP: adenosine triphosphate
N-BPs: nitrogen containing BP
IV: intravenous
PEG: polyethylene glycol
NIR: near-infrared
NPs: nanoparticles
BP NPs: poly(ethylene glycol) BP NPs
MA-PEG-BP: methacrylate-PEG-BP
APMA: 3-Aminopropyl)mathacrylamide
TTEGDA: tetra ethylene glycol diacrylate
PPS: potassium persulfate
PEG-NHS: O-[(N-Succinimidyl) succinyl-aminoethyl-O’-methylpolyethylene glycol
PVP: polyvinylpyrrolidone
NaOH: sodium hydroxide
HCl: hydrochloric acid
DMF: N,N-dimethylformamide
i-PrOH: isopropanol
THF: Tetrahydrofuran
NHS-PEG-NHS: O,O′-Bis[2-(N-succinimidyl-succinylamino)ethyl] polyethylene glycol
BB: sodium bicarbonate
rt: room temperature
